# Short and long-term genome stability analysis of prokaryotic genomes

**DOI:** 10.1186/1471-2164-14-309

**Published:** 2013-05-08

**Authors:** Matteo Brilli, Pietro Liò, Vincent Lacroix, Marie-France Sagot

**Affiliations:** 1 INRIA, Grenoble Rhône-Alpes, Lyon, France; 2 Laboratoire de Biométrie et Biologie Évolutive, Universitè Lyon 1 UMR CNRS 5558, Lyon, France; 3 Computer Laboratory, University of Cambridge, 15 JJ Thompson Avenue, Cambridge, CB3 0FD, UK; 4 Present address: Fondazione Edmund Mach/CRI - Functional genomics - Via Mach 1, 38010 San Michele all’Adige, Trento, Italy

## Abstract

**Background:**

Gene organization dynamics is actively studied because it provides useful evolutionary information, makes functional annotation easier and often enables to characterize pathogens. There is therefore a strong interest in understanding the variability of this trait and the possible correlations with life-style. Two kinds of events affect genome organization: on one hand translocations and recombinations change the relative position of genes shared by two genomes (i.e. the *backbone* gene order); on the other, insertions and deletions leave the backbone gene order unchanged but they alter the gene neighborhoods by breaking the syntenic regions. A complete picture about genome organization evolution therefore requires to account for both kinds of events.

**Results:**

We developed an approach where we model chromosomes as graphs on which we compute different stability estimators; we consider genome rearrangements as well as the effect of gene insertions and deletions. In a first part of the paper, we fit a measure of backbone gene order conservation (hereinafter called backbone stability) against phylogenetic distance for over 3000 genome comparisons, improving existing models for the divergence in time of backbone stability. Intra- and inter-specific comparisons were treated separately to focus on different time-scales. The use of multiple genomes of a same species allowed to identify genomes with diverging gene order with respect to their conspecific. The inter-species analysis indicates that pathogens are more often unstable with respect to non-pathogens. In a second part of the text, we show that in pathogens, gene content dynamics (insertions and deletions) have a much more dramatic effect on genome organization stability than backbone rearrangements.

**Conclusion:**

In this work, we studied genome organization divergence taking into account the contribution of both genome order rearrangements and genome content dynamics. By studying species with multiple sequenced genomes available, we were able to explore genome organization stability at different time-scales and to find significant differences for pathogen and non-pathogen species. The output of our framework also allows to identify the conserved gene clusters and/or partial occurrences thereof, making possible to explore how gene clusters assembled during evolution.

## Background

Genome dynamics are mainly studied in relation to gene content, with several evolutionary models adapted to the problem, such as for instance *birth-death and transfer* models [1-4]. These approaches contributed to the development of the concepts of *core* and *accessory* genome: genes shared by all genomes of a species constitute the core, whereas accessory genes are present in a subset of the genomes. The maintenance of many prokaryotic genes is influenced by ecology, and accessory genes often carry information about peculiar adaptations (e.g.[5-13]). It is therefore conceivable that the fitness associated with a given genome organization depends in a similar way on the life-style of an organism: gene clusters may be transferred or assembled/disassembled, providing information on the selective pressures acting on peculiar gene associations in different ecological scenarios. Specific chromosome organizations (e.g. operons, genomic islands or larger aggregates) can be preferred by evolution, for instance through the selection of a given distribution of genes relative to the origin of replication or a specific pattern of gene co-expression. Chromosome rearrangements antagonize the selective features of the organization of bacterial genomes so that a trade-off is reached integrating the selective advantage of specific gene associations in an evolutionary and ecological context. Besides their destructive role, rearrangements allow exploring alternative gene associations and are therefore of paramount importance for genome evolution. Different rates of genome rearrangements characterize different ecological constraints, making the study of genome organization stability particularly interesting. Pathogens are for instance under periodic selection e.g. imposed by the immune system or drug treatments, and it was suggested that they have genomes with plastic organization [14]. However, the transient nature of these selective pressures on the long evolutionary scale must be taken into account to identify stable and unstable genomes and gene associations: if historical niches do not reflect modern environmental associations, merging comparisons spanning millions or billions of years can obscure the true relationship with life-style: genes forming an operon in *Escherichia coli* can be scattered in other species [15-19], indicating that the tendency of particular genes to stay close on the genome is subject to evolutionary change, like all biological properties.

Gene order analysis is also an important tool in comparative and functional genomics since conserved gene clusters often comprise genes with related functions [20-26]. The importance of gene clustering in evolution has started being recognized for eukaryotes too [27-31].

Rocha [32] focused on the divergence of core genes organization with respect to phylogenetic distance for over one hundred genomes of different taxa. Stability was quantified as the frequency at which contiguous genes in a genome are contiguous in another. Accessory genes were deleted from the ordered gene lists to be compared and the two flanking core genes were then considered to be contiguous. We will indicate this approach as *backbone* stability analysis since it focuses only on core genes order. The best fit between this backbone stability (BS) estimator and phylogenetic distance was obtained with the following model:

(1) $${\hat{\text{BS}}}_{\text{Rocha}}=\frac{p_{f}^{t}+p_{s}^{t}}{2},$$

where *p*_*f*_ and *p*_*s*_ correspond to the probability of splitting contiguous genes for fast and slow rearranging gene pairs, respectively. This model is a special case of Eq. 2 when the genome is partitioned into two equally populated categories of fast and slow rearranging gene pairs:

(2) $${\hat{\text{BS}}}_{\text{Rocha}}=\frac{n_{f}\cdot p_{f}^{t}+n_{s}\cdot p_{s}^{t}}{N},$$

where *N*=*n*_*f*_+*n*_*s*_ is the number of pairs of genes in the genome. A similar strategy was previously used by Huynen et al. [19] leading to the same conclusions. Tamames et al. [18] used a different strategy and proposed a sigmoid relationship between genome organization conservation and phylogenetic distance. In this case the authors identified orthologous genes between pairs of genomes, extracted genome regions with conserved gene order and calculated their stability estimator as the fraction of genes in conserved runs with respect to the number of genes. In this case, accessory genes help defining the borders of the conserved regions.

Previous works therefore express different views on how genome organization changes in time which could be ascribed to the genomes selected for the comparisons or to differences in the analytical methods. Specifically, the way the insertion/deletion of accessory genes is addressed is relevant in this context since genome organization divergence is the resultof the interplay between genome rearrangements (i.e. translocations and recombinations) and gene content dynamics (insertions and deletions). The latter do not change the relative order of core genes, and are consequently neglected in backbone stability analyses. By taking them into account we can identify evolutionarily persistent gene associations but it is difficult to discern between the contribution of genome rearrangements and gene content dynamics to genome organization divergence. Based on this, a complete picture on genome organization evolution clearly requires considering the information coming from both core gene order (backbone stability) and insertions/deletions of accessory genes (genome organization stability).

To fulfill this task, we implemented a graph-based framework to study in depth the stability of prokaryotic genomes and applied it to a selected dataset of genomes. We improved Eq. 1 for backbone stability in time, and then we compared the fit of the new and several other models to the data. Using the fitted model, we studied genome backbone stability within and between bacterial species to better understand genome organization dynamics on the short and the long evolutionary time. The relationship between backbone stability (BS) and genome organization stability (GOS) provided information about the importance of genome organization rearrangements and gene content dynamics for genome evolution in different species. A comparison between GOS and genome fluidity [33] allowed to summarize the variability in the size of accessory gene clusters in different species highlighting differences between pathogens and non-pathogens. An additional output of our approach is the phylogenetic distribution of conserved gene clusters in the genomes under analysis, which provides useful evolutionary insights on how they are distributed and assembled.

We discuss our results in an ecological perspective where the life-style of the species under analysis is taken into account to explain the properties of the corresponding genomes.

## Results and discussion

### Strategy and definitions

**Figure 1:**
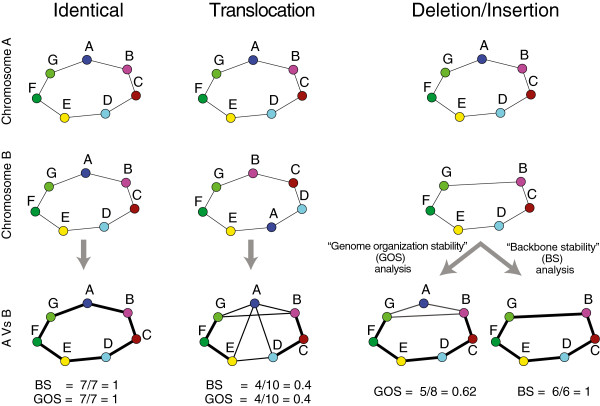
**Backbone and genome organization stability.** The scheme shows how we encode and combine individual chromosomes for their comparison; nodes (A to G) represent genes; edges between nodes indicate that the genes are contiguous on the chromosome. The figure provides examples illustrating the difference between backbone and genome organization stability. Since we deal with pairwise comparisons, we are not able to differentiate deletions from insertions. Thick edges correspond to conserved gene neighborhoods (present in both genomes). The two stability measures are identical when there are no accessory genes (first two colums).

**Figure 2:**
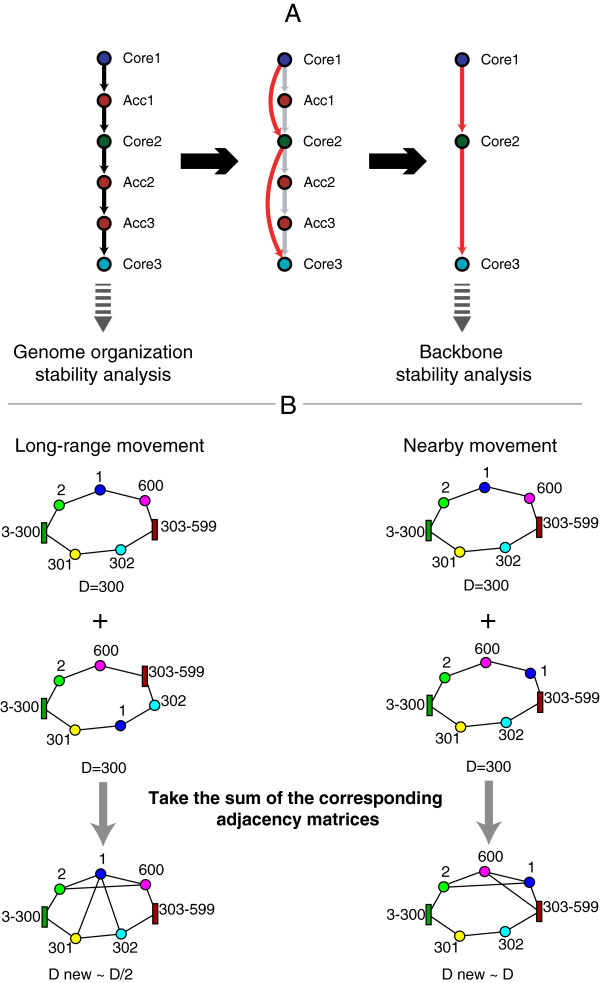
**Methods.A)** Graph compression to eliminate accessory genes in backbone stability calculation; **B)** Two hypothetical genome comparisons to illustrate the effect of rearrangements on the diameter of the graph. The chromosomes under comparison have 600 genes each (two clusters of genes are compressed into rectangular nodes). In the comparisons, the two chromosomes differ in the position of gene 1, they have the same number of genes, and therefore the same diameter (*D*). Let us focus on the networks obtained after combining the chromosomes under analysis: as a consequence of the different positions of gene 1 in the two chromosomes under comparison, new edges are formed between them, and this affects the diameter of the combined graph. When rearrangements involve distant loci (left), there is a strong effect on the diameter (in the example, it halves). On the converse, rearrangements between nearby loci (right) have a weak effect on the diameter.

Our strategy can be schematized into three major steps: *Orthologous mapping*, *Gene neighborhood network reconstruction and comparison* and *Stability assessment*. In the first step all the proteins from a group of genomes are classified into orthology groups. This is a critical step whose output affects the entire strategy; our implementation is explained in the *Methods* section. In the second step genomes are translated into adjacency matrices exploiting tables of gene coordinates. The adjacency matrix encodes a network where a node (i.e. a gene) is connected to the previous and to the next one on the chromosome, so that for a circular chromosome we obtain a ring of nodes. We called the network for a given strain *Genome Specific Neighborhood network* (GSN) (see the example in Figure 1, first two lines of drawings). The orthology mapping allows to encode all the chromosomes in the same way (e.g. an ortholog in different genomes has the same position in all adjacency matrices). The comparison between different chromosomes is simply done by summing the adjacency matrices corresponding to the two genomes (the GSNs), obtaining a weighted network (the *General Gene Network*, GGN) with two kinds of edges: conserved, with a value of 2, that are present in both networks, and non-conserved, with value 1, that are present in only one of the two networks (Figure 1). This network is the input for the calculation of GOS stability and diameter. For BS analysis, we add a *Compression* step before the comparison, so that we only consider core genes (Figure 2A and Figure 1C). The BS coefficient between genome *i* and genome *j* is defined in the following way:

(3) $$BS_{\text{ij}}=\frac{N_{\text{ij}}^{\text{cn}}}{N_{\text{ij}}^{\text{tot}}},$$

where $N_{\text{ij}}^{\text{cn}}$ and $N_{\text{ij}}^{\text{tot}}$ are the number of conserved and total edges (conserved + non conserved) in the comparison between genome *i* and genome *j*, respectively. It follows that *BS*_*ij*_∈ [0,1], and thanks to the compression step it measures how much conserved is the *core* gene order in genome *i* with respect to genome *j* (see *Methods* and Figure 2A). Broadly speaking, the stability of two genomes with very similar core gene order is close to one, even if there are many accessory genes, while it diminishes when divergence in gene order increases, becoming zero when genes are organized in completely different ways. Genome organization stability (GOS) is instead calculated by taking into account the presence of accessory genes:

(4) $$\text{GO}S_{\text{ij}}=\frac{N_{\text{ij}}^{\text{cn}}}{N_{\text{ij}}^{\text{cc}}+\frac{N_{\text{ij}}^{\text{ac}}}{2}},$$

where at the denominator we only consider edges connecting core (*N*^*cc*^) and core with accessory genes (*N*^*ac*^) to reduce the effect of the size of accessory DNA fragments. If the denominator were simply *N*^*tot*^ as in Eq. 3, any insertion of large gene clusters would strongly affect GOS, while what matters is the number of times a gene or a group of genes is inserted within core genes. GOS therefore integrates stability in terms of genome rearrangements and in terms of neighborhoods broken by the insertion/deletion of accessory genes; we call attention to the fact that GOS is very similar to the genome fluidity defined by [33] to study genome content dynamics:

(5) $$\varphi_{\text{ij}}=\frac{N_{\text{ij}}^{\text{acc}}}{2N_{\text{ij}}^{\text{core}}+N_{\text{ij}}^{\text{acc}}},$$

where $N_{\text{ij}}^{\text{acc}}$ ($N_{\text{ij}}^{\text{core}}$) is the number of accessory (core) genes for the comparison between genome *i* and *j*; *φ* is therefore a measure of gene content variability. Considered from a different perspective, 1-*φ* is a measure of gene content stability:

(6) $$\sigma_{\text{ij}}=1-\varphi_{\text{ij}}=\frac{N_{\text{ij}}^{\text{core}}}{N_{\text{ij}}^{\text{core}}+\frac{N_{\text{ij}}^{\text{acc}}}{2}}.$$

The input for computing GOS is the same as for diameter calculation. The latter is the longest shortest path connecting any two nodes in a network. The shortest path between two nodes in a graph is defined as the path with the minimum number of edges between them. The diameter can be calculated in different ways; we use Johnson’s method [34] implemented in the Matlab library MatlabBGL [35]. We propose to use the diameter as an alternative stability measure because it allows to consider accessory genes and to convey additional information to the previous measures. As shown by Watts and Strogatz [36], the simple rewiring of a small fraction of the links in a regular lattice results in a sudden lowering of the diameter of the graph; similarly, when the position of a gene changes between different genomes, the diameter of the corresponding GGN shrinks (Figure 2B). It follows that the diameter is inversely related to the stability of the genomes.

The GGN can be obtained summing any number of adjacency matrices: if the edge values of the GGN are normalized by the number of genomes under comparison we obtain a weighted network with edge values corresponding to the fraction of times a given gene is found close to another one in these genomes. This new matrix allows a rapid extraction of gene clusters present in more than *α%* of the genomes by removing edges with a value under the threshold and collecting the induced connected components. To this purpose, we use the Dulmage-Mendelsohn decomposition in Matlab.

### Simulation of neutral gene order evolution

**Figure 3:**
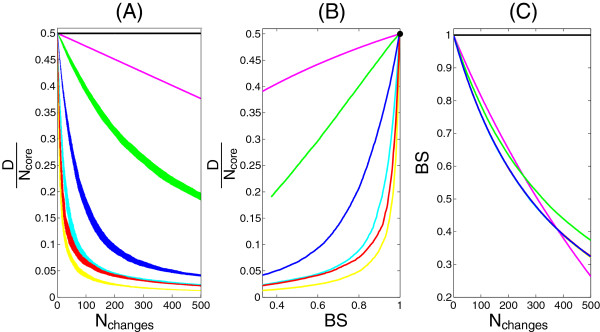
**Stability measures.** Simulations illustrating the relationship between the diameter and genome stability. The simulations start from an ancestor genome of 2000 genes arranged in a circular chromosome. At each step of the simulation one gene is picked at random, moved elsewhere in the genome according to different models and the new chromosome is compared to the ancestor. We simulated different evolutionary models, plotted in different colors: yellow: evolutionary model with random rearrangements; magenta: model with only deletions; black: deletions and graph compression. All other colors correspond to *local* rearrangements where the new position is sampled from a normal distribution N(μ,σ): cyan: N(100,5); red: N(200,5); Blue N(30,5); Green N(5,5). **A)** Relationship between the diameter, normalized by the number of core genes, and the number of gene movements after separation from the ancestor chromosome. In this panel, we also show the standard deviation for different simulations (thickness of the series). **B)** Relationship between backbone stability and normalized diameter. The relationship appears to be the inverse of that in **(A)** suggesting **(C)** an almost linear relationship between the number of rearrangements and backbone stability. The relationship of the stability with the number of changes does not contain information about the pattern of gene translocation while the diameter is markedly different for local or global gene movements.

In order to provide an intuitive understanding of the stability measures we used throughout this paper, we first performed simulations. The starting point of each simulation is a reference chromosome of 2000 genes. An exploratory analysis revealed that the relationships reported in Figure 3 have constant shape irrespective of the size of the genome (data not shown) and allowed to define the number of evolutionary steps to be performed. At each of 500 steps, one random gene is moved elsewhere on the chromosome and the resulting graph is compared to the reference (see *Gene Neighborhood Network reconstruction*) for assessing stability and measuring the diameter. In practice, the experiment simulates the divergence of a strain from its ancestor. We usedtwo models for gene translocations: in one case the target position on the chromosome is random; in the second case we model local translocations, with the new position given by *p*_*new*_=*p*_*old*_±*a*, where a∼N(μ,5) (the positive or negative sign are equiprobable). In this model, genes tend to move at an average distance of *μ* genesfrom the original location; we tested *μ*= [5,30,100,200]. We also added a genome evolutionary model based on gene insertions/deletions only. In Figure 3, we show the average values over 100 different simulations. The diameter appeared to be strongly affected by the very first gene translocations (Figure 3A). The effect is attenuated for a large number of changes. The *random* and *local* simulations are well separated. The diameter is a good genome stability measure in the short evolutionary time because its relationship with the number of rearrangements is very steep for short distances or high stability values (Figure 3B). The evolutionary model with only insertions/deletions predicts a linear relationship of the diameter with both the number of rearrangements and BS. The relationship of backbone stability with the number of rearrangements is almost linear (Figure 3C), justifying the use of thisstability measure. All models have the same slope here while the different patterns of genome rearrangements are clearly distinguishable using the diameter. In conclusion, both BS and the diameter of the network are strongly correlated with the number of gene translocations in our simulations.

### Backbone stability analysis

#### Modeling stability in time

Several models describing the divergence of gene order in evolution have been proposed [18,32]; in Table 1 we summarize them and we add three new models fortesting. The first one, (*Hill* in Table 1), is a sigmoid function with easily interpretable parameters: *k*, the *activation coefficient*, corresponds to the *x* value at which the function takes value 1/2 (half-maximum) and *n* determines the steepness of the shift from high to low levels (the *degree of cooperativeness*). We also derived two generalizations of the model used by Rocha (Eq.1 and [32]): we relaxed the assumption about the partition of a genome into two equally populated groups of fast and slow rearranging gene pairs in the following way:

(7) $$\hat{\text{BS}}=f_{s}p_{s}^{x}+(1-f_{s})p_{f}^{x},$$

**Table 1:** Model fitting results

**Name**	**Tested functions**	**N params**	**Adj.*R***^**2**^	**AIC**	**Akaike weight**	**SSE**
*Rocha*+3*p*	$\text{BS}=f_{i}+(1-f_{i}-f_{\text{ff}})p_{f}^{x}$	3	0.897	-12931	9.99E-01	46.1395
*Exp*	*BS*=*a*+*e*^*bx*^	2	0.895	-12910	2.86E-05	46.4842
*Rocha*+2*p*	$\text{BS}=f_{i}+(1-f_{i})p_{f}^{x}$	2	0.895	-12898	7.53E-08	46.6638
*Rocha*	$\text{BS}=\frac{p_{f}^{x}+p_{s}^{x}}{2}$	2	0.894	-12870	7.86E-14	47.0838
*Hill*	$\text{BS}=\frac{k^{n}}{k^{n}+x^{n}}$	2	0.892	-12785	3.05E-32	48.3971
*Tamames*	$\text{BS}=\frac{2}{1+e^{a\;x}}$	2	0.891	-12765	9.29E-37	48.7567

In the table, *a*, *b*, *k*, *n*, *f*_*f*_, *f*_*i*_, *f*_*ff*_, *p*_*f*_ and *p*_*s*_ are fitted parameters and *x* corresponds to the phylogenetic distance. Values are ordered by increasing AIC value. Models are fitted to the results obtained with the compressed networks.

with *p*_*s*_,*p*_*f*_,*f*_*s*_∈[0, 1]; *f*_*s*_ (1-*f*_*s*_) is the fraction of slowly (fast) rearranging gene pairs. The fitting performed with this formula returned a parameter *p*_*s*_ fixed at 1 by the algorithm; this allowed us to reduce the model (Eq. *Rocha*+2*p* in Table 1):

(8) $$\hat{\text{BS}}=f_{i}+(1-f_{i})p^{x},$$

Following this model, a certain fraction *f*_*i*_ of edges is invariant, whereas the remaining are maintained with probability *p*. A further extension of the model considered the presence of a third category of very labile gene pairs, such that the probability of conservation is negligible (*p*_*ff*_=0) at the time resolution of the model (Eq. *Rocha*+3*p* in Table 1):

(9) $$\hat{\text{BS}}=f_{i}+(1-f_{i}-f_{\text{ff}})p^{x},$$

Results of the non linear fitting are reported in Table 1. The *Rocha*+3*p* model has the minimum AIC and best explains the data. The Akaike weights indicate that its probability of being the correct model is much higher than for the others. The first observation on estimated parameters is that *p*_*f*_ takes the same value in the two *Rocha* models, around 0.24, and so does the *f*_*f*_ fraction, indicating that about 95% of the gene pairs change quite frequently.Following the *Rocha*+3*p* model, a small fraction of edges (*f*_*ff*_=0.02) changes very fast. These might involve transposases and other mobile elements. It should be noticed that the original *Rocha* model gives *p*_*f*_=0.17 and *p*_*s*_=0.37, not too far from what we obtained here.

#### Intra-species stability analysis

**Figure 4:**
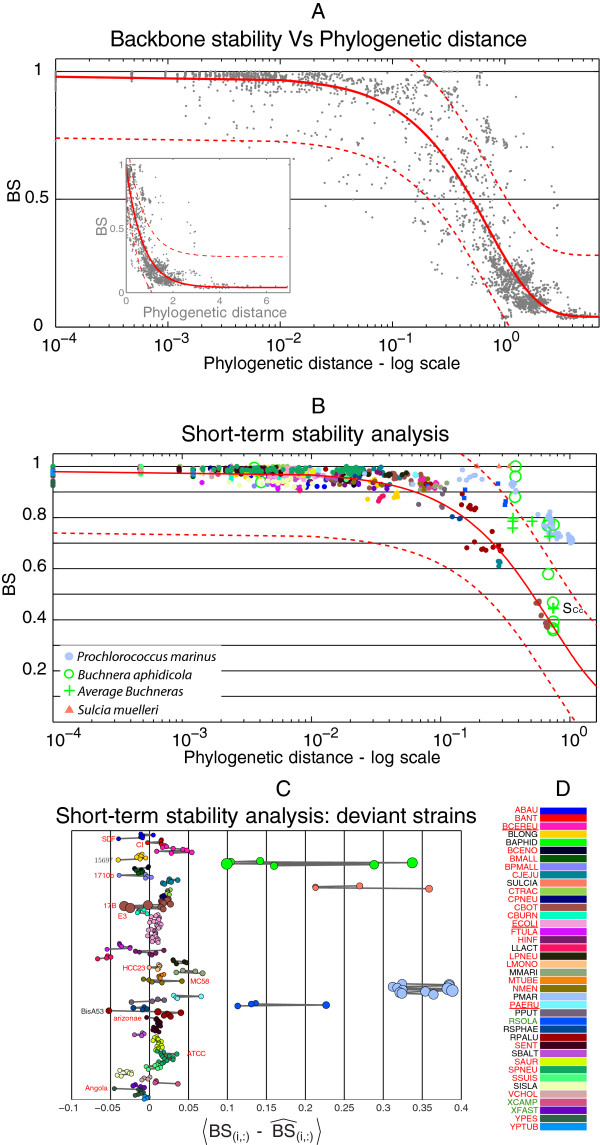
**Intra-species backbone stability analysis.A** Data used for model fitting and the prediction and confidence intervals (continuous and dashed red lines) of the best model (*Rocha*+3*p* in the text). In the main plot, the phylogenetic distance is in log scale for clarity (see inset for original values). **B** Intra-species comparisons. The endosymbionts *Sulcia* and *Buchnera* have very stable genomes, but the latter has more variability in gene order. **C** Residuals with respect to the best model for each strain. Strains more than 2 standard deviations from the average residual within a species are highlighted. **D** Color code for species. Species abbreviations are color coded in the following way: red, pathogens; underlined red, species comprising both pathogens and non-pathogens; green, plant pathogens; black, non-pathogens.

In Figure 4 we plot the backbone stability for all intra-species comparisons; genomes above the model predictions are more stable than expected: *Sulcia muelleri*, *Buchnera aphidicola* and *Prochlorococcus marinus* are the most evident cases. In the case of *Sulcia*, and despite the large phylogenetic distance dividing these genomes, the backbone is almost completely conserved. The age of the symbiosis between *Sulcia* and its host (260 Mya [37]) might explain this high stability. Moreover, the fact that these strains have completely conserved gene orders suggests that they maintained an intact chromosome structure since the time of their separation. In agreement with available information, we also detected increased genome stability for the other endosymbiont of our dataset, *B. aphidicola*. However, the conservation of gene organization is not marked as in *Sulcia* and moreover we noticed that *B. aphidicola* Cc diverges with respect to the other strains (as indicated by its average stability value, *BS*_*Cc*_ in Figure 4). This strain has peculiar features with respect to other *Buchnera*s: its host (the aphid *Cinara cedri*) harbors two additional symbionts that are as abundant as *Buchnera*, suggesting the possibility of *metabolic* replacement [38]. It seems therefore plausible that the presence of other symbionts has relaxed the selection on some of the activities provided by *Buchnera*, promoting their loss [38], and on some of the neighborhoods as suggested by our analysis.

*Prochlorococcus marinus* was identified as unstable in Rocha [32] on the basis of the comparisons of three *P. marinus* and five other Cyanobacteria. This discrepancy derives from the different phylogenetic ranges of the comparisons here and in [32]. By looking more in detail at the behavior of two additional Cyanobacteria present in our *genus* dataset (see Methods and Additional file 1), we found that the *P. marinus* genomes have indeed higher stability (Additional file 2). This reinforces the idea that by considering multiple genomes of the same species we can define the behavior of each group of organisms in a better way. Our analysis moreover shows that *P. marinus* is relatively unstable on the long-term, in agreement with [32].

The intra-species analysis shows that all species have more or less stable gene organization. To identify aberrant genomes within a species, we obtained Z-scores for the residuals of each genome with respect to its conspecific and the model predictions (Figure 4C); genomes with the largest deviation from the mean are discussed below in the light of previous reports about genome organization. Despite the only marginal sequence divergence within the analyzed isolates, strain Angola appeared to be highly rearranged with respect to the remaining *Y. pestis* genomes. A further analysis indicated that this strain shares 92% of the gene pairs with *Y. pseudotubercolosis* IP_32953, which is almost the same as with the other *Y. pestis* genomes. The similarity in gene order among the other *Y. pestis* genomes is instead much higher (they share on average 99% of the gene pairs) as it is higher the similarity of these strains with *Y. pseudotubercolosis* IP_32953 (about 96% of the gene pairs are in common). This suggests that strain Angola experienced a period of intense reorganization after the separation from *Y. pseudotubercolosis* and independently from other *Y. pestis* strains, in agreement with the high degree of intrachromosomal rearrangements detected in a dedicated comparative analysis [39].

The *Bacillus anthracis* CI genome was previously analyzed by a comparative genomics approach leading to the conclusion that it has evolved from a *B. cereus* strain and established a *B. anthracis* lifestyle [40]. This is also reflected in a markedly different gene organization with respect to the other genomes of the *anthracis* clade, as it appears from Figure 4C.

Sela et al. [41] found an abundance of mobile genetic elements in the genome of *Bifidobacterium longum* ATCC15697 relative to other sequenced bifidobacteria, a feature positively affecting rearrangement frequencies [42]. *Acinetobacter baumannii* is the source of numerous nosocomial infections in humans and is often multidrug resistant. Comparative genomics revealed that strain SDF is highly divergent from strains ADP1 and AYE and that it harbors over 400 insertion sequences, much more than other strains [43]. This, along with our stability analysis, suggests that this strain is undergoing an intense rearrangement of gene order, perhaps as a consequence of the adaptation to the human host or the new challenges imposed by drug treatment.

#### Inter-species stability analysis

For the inter-species analysis, we selected one stable genome *per* species on the basis of the previous analysis and we compared them all against all. Genomes more stable than expected at such large phylogenetic distances were those of *B. aphidicola*, *S. muelleri* and *Coxiella burnetii* (not shown). The latter is a widespread bacterium causing Q fever in humans whereas it does not normally cause overt disease in its reservoirs (cattle, sheep, goat). Its genome stability seems to be in contrast with the phenotype in humans but it agrees with its obligate intracellular life-style. It was indeed shown to have rearranged genomes with large syntenic blocks [44].

The presence of the two endosymbionts suggests that rearrangements played a minor role in their genome evolution, indicating that these endosymbionts diverged from their ancestors by eliminating superfluous genes and joining the remaining without much rearrangements.

**Figure 5:**
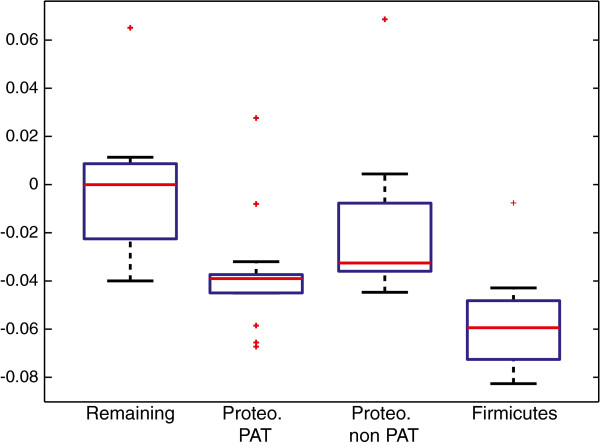
**Inter-species analysis.** Boxplot of residuals of empirical data with respect to model predictions for inter-species comparisons. Firmicutes have significantly smaller residuals than other taxonomic groups (*p*=0.0022) and are mainly pathogens. A weak but significant association of pathogenesis with instability exists in the Proteobacteria (*p*=0.042).

Some of the genomes that were stable following the intra-species analysis showed here instability. We observed that 8/9 of the genomes with highest instability in these comparisons belong to animal pathogens, whereas this category represents about 62% of the genomes in our dataset; however, there is a significant association of instability with the taxonomic affiliation of the genomes, since 6 of these genomes come from the Firmicutes. We checked if Firmicutes tend to be less stable than other genomes and this is indeed the case (Figure 5, *p*=0.0022 in a Wilcoxon rank sum test), therefore the observed difference between pathogens and non-pathogens may be related to the Firmicutes in our dataset being mostly pathogens. To clarify this point, we focused on Proteobacteria by testing for equality of the median residuals of pathogens and non-pathogens; we obtained a weak but significant difference (Figure 5, *p*=0.042, when *Buchnera* is not included in the non-pathogens). Our analysis therefore indicates that pathogens tend to be less stable than non-pathogens and that they experienced past periods of rearrangements, plausibly during adaptation to their new life style. Since these genomes are not particularly unstable on the short evolutionary time, instability was transitory, highlighting the importance of considering multiple time-scales for these comparisons. It should be noticed however that these signals may be related to other taxonomic effects, as also noticed before [32], and that can be avoided only with much larger and balanced datasets.

### Genome organization stability

In the previous section, we analyzed what we called backbone stability. In that case, we neglected the effect of accessory genes to highlight gene movements along the chromosome, and we observed a general stability of genomes on the short evolutionary time. However, the stability of a genome is the consequence of two processes: genome order rearrangements and gene content dynamics. In this section, we focus on genome organization stability using the diameter and the stability measure defined in Eq. 4 where insertions and deletions are also considered.

#### Diameter is a proxy for genome stability

**Figure 6:**
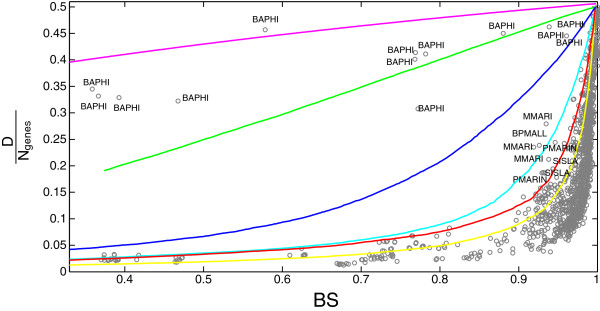
**Diameter of the gene neighborhood networks.** The relationship between backbone stability and diameter for simulations (lines, colors as in Figure 3) and intra-species comparisons (dots). We find that most genomes evolve by moving genes at large distance from the original position; *Buchnera* has a markedly different behavior, with data points mainly located in between the evolutionary model with only deletions (magenta) and local rearrangements (green).

When chromosomes are compared following our strategy, the diameter can be another useful proxy for GOS, especially when the distances are short. In Figure 6, experimental data points are mostly located to the right of the simulations, suggesting that the majority of gene translocations involve distant loci. All experimental points located very far from the bulk of the data correspond to comparisons involving *Buchnera*. The genomes of this species show a very anomalous relationship of the diameter with stability, in-between a pattern of only deletions and local gene movements. Since the *B. aphidicola* genomes evolved mainly by deleting genes from an *E. coli*-like ancestral genome [45], this analysis, together with the previous ones, strongly suggest that the process is still ongoing: different *Buchnera* strains are independently deleting genes as a consequence of the selective pressure experienced in specific hosts. It is an open question if they will stabilize on similar gene contents, or if they will show signatures of the different metabolic pressures experienced in different hosts, as suggested by the peculiarities of *B. aphidicola Cc*. The genomes of the other endosymbiont, *Sulcia*, have instead large and almost constant diameters, in agreement with the extreme stability of their genome backbone.

#### Most gene associations are rapidly erased

**Figure 7:**
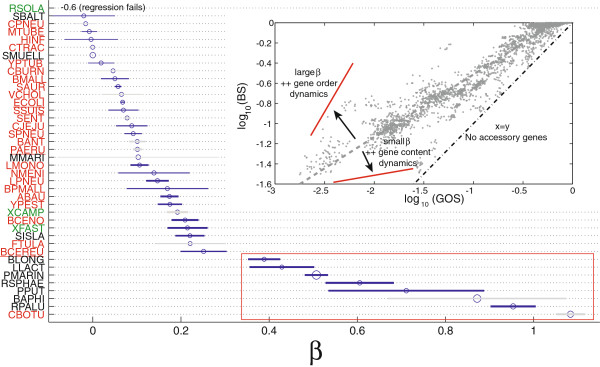
**Backbone stability Vs Genome organization stability.** Inset: the relationship between BS and GOS for all comparisons (intra- and inter-species). Main panel: markers correspond to the *β* coefficients of the regression model *BS*=*α*+*βGOS* for intra-species comparisons and the line their standard errors. The larger the *β*, the greater the importance of gene order rearrangements for genome organization evolution. A small coefficient indicates that genome content dynamics has a larger impact on genome evolution. Size of the marker is proportional to the average phylogenetic distance within a species. Species for which the quality of the regression is not good are in gray (see Additional file 3). The largest coefficients correspond to free-living species.

The relationship between backbone stability (Eq. 3, BS) and genome organization stability (Eq. 4, GOS) provides insights about the relative importance of gene order rearrangements and genome content dynamics for genome organization divergence: BS is affected only by rearrangements, while GOS is a combination of the two. The two stability measures are identical only when there are no accessory genes. At short phylogenetic distances, most of the neighborhoods are broken by genome content dynamics: (BS is very close to 1 while GOS falls from 1 to 0.4-0.5, data not shown), hence genome order rearrangements have a minor effect on genome organization divergence at these phylogenetic distances. Since the two variables are linearly related in intra-species comparisons (Additional file 3), we use the slope of this relationship as a measure of the importance of the two processes for genome organization evolution: small coefficients correspond to a larger contribution of gene content dynamics, whereas larger ones imply more rearrangements (see the inset in Figure 7). We built linear regression models for each species separately using the intra-species comparisons (Additional file 3). We show the sorted coefficients and their standard error in Figure 7. Most non-pathogens are grouped at the bottom of the plot, corresponding to larger coefficients. The probability of sampling 6 non-pathogens species in our dataset can be calculated, giving *p*=2.8*E*-04 (even by excluding *Buchnera*). The only pathogen within these species is *Clostridium botulinum* for which however there are two groups of points biasing the regression estimate (Additional file 3); even by including this species the result is still highly significant (*p*=0.001). This suggests that at short phylogenetic distances, non-pathogens have slower gene content dynamics than pathogens, with rearrangements playing a major role in genome organization evolution.

#### Accessory components have widely different sizes

**Figure 8:**
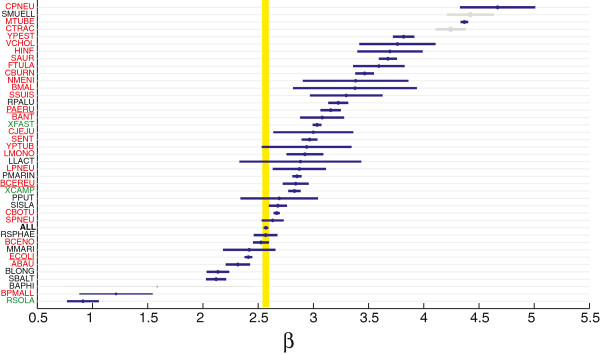
**Genome organization stability Vs Gene content stability.** Regression coefficients of the model *GOS*=*α*+*βσ*, where *σ*=1-*φ* and *φ* is the fluidity of genome content defined in [33] and Eq. 5. The yellow line corresponds to the coefficient obtained when all data points are used together (the tickness corresponds to its standard error). Species for which the regression is not good are in gray (see Additional file 4). Marker size is proportional to the intra-species phylogenetic distance.

Since GOS is affected by rearrangements of core genes and by the number of accessory gene insertions/deletions whereas genomic fluidity (Eq. 6 and [33]) is affected by the number of accessory genes, the relationship between the two indicates how strong is the effect of adding accessory genes on GOS. In other words, this relationship is informative on the size of accessory components: when *N* accessory genes integrate in the genome one by one, their effect on GOS is maximal because *N* integration events interrupt *N* core-core neighborhoods; the genomic fluidity is affected similarly. On the other extreme, i.e. when *N* accessory genes are inserted as a large gene cluster, the effect on GOS is small because only one core-core neighborhood is broken; the genomic fluidity is instead unchanged with respect to the previous example. The relationship between GOS (Eq. 4) and *σ* (Eq. 6) is therefore informative about the typical length of accessory gene clusters. The relationship between the log transformed variables is linear (Additional file 4), allowing an easy comparison of the behavior of each species and the whole dataset by focusing on the regression coefficients (*β*) of the model *GOS*=*α*+*βσ*. In particular, given e.g. *β*=*x* for the whole intra-species dataset, the species with *β*<*x* are characterized by larger accessory gene clusters whereas those with *β*>*x* by smaller accessory clusters. Our results (Figure 8) suggest that there are wide differences in the size of accessory gene clusters, with *Chlamydophila pneumoniae*, *Mycobacterium tubercolosis* and *Yersinia pestis* having the smaller accessory gene clusters, whereas *Ralstonia solanacearum*, *Burkholderia pseudomallei*, *Shewanella baltica* and *Bifidobacterium longum* have the largest ones. Several species in the plot (in gray), have only a few accessory genes, making impossible to get the right parameter estimates, comprising the endosymbionts of our dataset and *Chlamydia trachomatis*, a pathogen with an obligate intracellular life-style. The 10 species with the largest coefficient are significantly enriched in pathogen species (*p*=0.024) suggesting that pathogens tend to modify their genome content by gaining and removing blocks of genes that are on average smaller than for non-pathogens.

**Figure 9:**
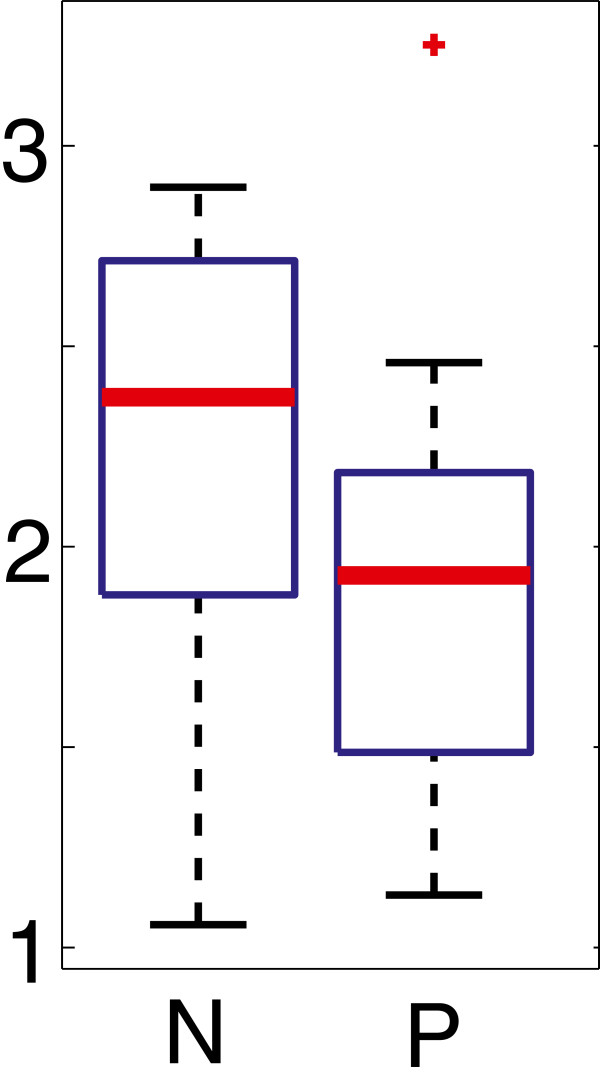
**Size of Singleton gene clusters.** Distribution of the average singleton gene cluster size in pathogens (P) and free-living non-pathogens (N).

To explore this issue more in detail, we investigated the distribution of the size of accessory gene clusters present in only one of the genomes within a species (*singleton gene clusters*), which are enriched in horizontally transferred genes [46-48]. We found a linear distribution in double logarithmic plots (Additional file 5): most of the clusters are therefore small but the probability of large clusters is greater than for a normal distribution with the same mean. By analysing the average size of the singleton clusters we find that the non-pathogens tend to have larger singleton components (there are 7 non-pathogens out of 10 species, *p*=0.003). A Wilcoxon rank sum test supported a separation of pathogens and non-pathogens based on the average size of singleton gene clusters (*p*=0.046) and the significance increased when further considering free-living non-pathogens only (*p*=0.003, Figure 9). When considering singleton gene clusters of at least 2 genes, the significance of this difference vanishes; pathogens have therefore more frequently isolated singletons, suggesting that differences may exist in the preferred way to acquire foreign genes.

#### Conserved gene clusters

**Figure 10:**
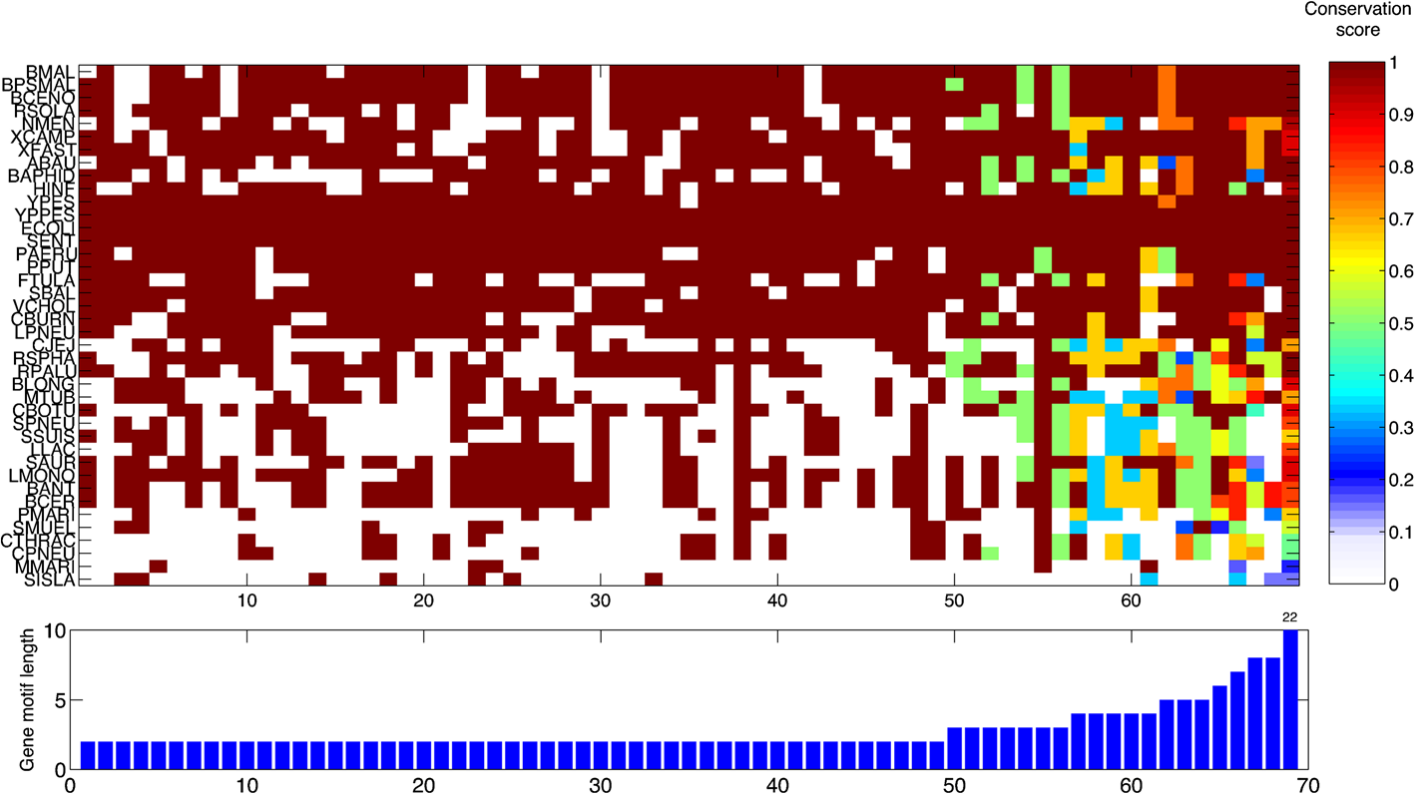
**Phylogenetic distribution of gene clusters.** Heatmap: columns correspond to gene clusters present in *E. coli* and conserved in at least 50% of the other genomes (rows). The value of each cell is the fraction of gene pairs in the gene cluster that are also present in a genome. Genomes are ordered on the basis of the phylogenetic distance with respect to *E. coli*. Bottom chart: length of the gene clusters.

The analysis on stability reveals a few stable gene associations even at large phylogenetic distances. To go further into the problem, one may wonder which are the genes involved in such associations, their function and phylogenetic distribution. Here we summarize an additional output of our framework and we focus on the gene clusters present in *Escherichia coli* and in at least 50% of the genomes of the inter-species dataset. We set this threshold to highlight gene clusters whose maintenance responds to widespread selective pressures. We obtained 69 gene clusters of 2 to 22 genes; only 8 gene clusters have more than 4 genes. We use the fraction of edges of the gene cluster that are present in the genome as a conservation score (*CS*), which also provides an indication about partial occurrences (e.g. *CS*=0.5 means that half of the gene pairs of the gene cluster are also present in the genome). We plot the results in Figure 10: clusters with 4 or more genes are often only partially conserved in other species, with a trend of increasing score towards *E. coli* and its closest relatives. Most of the genes of the conserved clusters form larger operons in *E. coli*, leading to hypothesize they might represent the building blocks of larger and eventually lineage specific gene clusters and operons.

There are 7 and 4 gene clusters that are also present in the Archaea *Sulfolobus islandicus* and *Methanococcus maripaludis*, respectively; among those only one is common (gene cluster 23, comprising two genes involved in tryptophan biosynthesis). These gene clusters code for interacting proteins and have metabolic roles (e.g. enzymes for tryptophan, arginine, leucine and pyrimidine nucleotides biosynthesis, glycine cleavage for serine biosynthesis), in addition to the Phe-tRNA synthetase subunits *α* and *β* (gene cluster 25). The only gene cluster present in the Archaea and comprising more than two genes codes for the PhoU regulator and three subunits of a phosphate transporter (cluster 61, partially conserved in *S. islandicus*), underlining the importance of this limiting nutrient across the prokaryotic kingdoms. The 3 gene clusters with the wider phylogenetic distribution code for three subunits of cytochrome oxydase (cluster 50), the elongation factor G plus two ribosomal proteins (cluster 53) and two ribosomal proteins (cluster 38). The functions encoded are in this case more housekeeping but similarly to the previous case, all of them encode interacting proteins. These results lead to the testable hypothesis about protein interaction being the major driving force for gene clustering, facilitating the initial assembly of gene clusters that are then combined to form larger gene aggregates during evolution.

## Conclusions

We studied the genome organization stability of 40 prokaryotic species for a total of 277 genomes, using an approach based on interpreting chromosomes as graphs. We focus on two different time-scales finding that at short phylogenetic distances, genomes are all quite stable besides life style; the use of multiple genomes of the same species allowed the identification of genomes with increased instability within a species, which are in majority from pathogens. Our results are in agreement with previous findings indicating that during adaptation to pathogenesis, several species experience phases of instability [49-51]. We confirm the high stability of endosymbiont genomes, adding moreover a few hints: *B. aphidicola* Cc has a deviant stability with respect to the other *Buchnera*s, plausibly because of its coexistence with other symbionts within the host [38]. The results show at the same time that *Sulcia muelleri* and *Buchnera aphidicola* differ concerning the stability of their genomes, with *Buchnera* having much more variability of both gene order and gene content. This suggests that *Sulcia* is more terminally differentiated, with a static backbone gene order and slow gene content dynamics.

The long term analysis allowed to identify those genomes that, although stable on the short time, are instead unstable on the long evolutionary time. As in the previous case, the genomes with increased instability were often from pathogens, indicating that at least some of them experienced instability periods during evolution while being quite stable today.

The comparison between backbone and genome organization stability for intra-species comparisons allowed to detect an important difference between pathogens and free-living non-pathogens: gene content dynamics plays a much more prominent role in the evolution of pathogen genomes, whereas free-living species tend to have slower gene content dynamics. We have moreover shown that non-pathogens tend to gain/delete fragments of the genome containing on average more genes than what is observed in pathogens.

Gene transfers play a fundamental role in genome evolution, therefore we focused on *singleton gene clusters*, that is gene clusters formed by genes present in only one of the genomes of a given species. It was shown that this category of genes is often enriched in xenologous genes, and this analysis may therefore inform about the size of transferred DNA fragments. We find that non-pathogens have a significantly larger mean size of singleton gene clusters. The statistical significance vanishes when the mean is calculated for gene clusters of at least two genes in length, indicating that the difference is not caused by larger clusters in the non-pathogens, but instead by a larger fraction of isolated singletons in pathogens.

Insertions and translocations in multiple genomes define the borders of evolutionary conserved gene clusters that can be rapidly extracted from our graphs by filtering the edges on the basis of the degree of conservation. Depending on the threshold and the organisms used, they can be seen as gene associations with different evolutionary success, and thus they may be related to more or less universal selective pressures. We show here that when focusing on gene clusters common to distantly related organisms, we mostly detect clusters encoding interacting proteins. This suggests that the main selective pressure towards gene clustering could be the co-localization of the synthesis of interacting partners, as it has been previously proposed on a limited number of genomes [18,52,53]. Even if only partial, our analysis also suggests that these conserved gene clusters may function as *nucleation* sites for the evolution of larger ones. Two lines of evidence support this view: most of the shorter clusters are known to be involved in longer operons in *E. coli* (e.g. tryptophan biosynthesis, clusters 23 and 24 and leucine biosynthesis, cluster 4), and larger gene clusters show high conservation in close *E. coli* relatives and only partial conservation in more distant ones.

## Methods

### Strategy and stability measures

Our strategy is briefly described at the beginning of the *Results* section, here we add some technical description.

#### Orthologous mapping

We classify orthologs with the BBH (“Bidirectional-Best-Hit”) criterion [54] by comparing all the proteins coming from a group of genomes at once. After an all-against-all blast, we build a similarity network where two proteins are connected if they are reciprocal best hits. We define a cluster in such a network as an *orthologous group* (OG). Since we require a one-to-one orthologous mapping for assigning unique gene neighborhoods to all genes in a genome, we systematically excluded all proteins belonging to an OG that contains multiple proteins from a same organism. When reconstructing the gene neighborhood graph, we skip the corresponding genes, i.e. the gene cluster *A*→*B*→*C*→*D*, in case *B* and *C* have been assigned to multiple orthologs group, becomes *A*→*D*. Removed genes correspond mainly to transposases present in multiple (almost) identical copies in most of the genomes under analysis (Additional file 6) and for which a one-to-one mapping is almost impossible without adding some information.

#### Gene neighborhood network reconstruction and comparison

The *gene neighborhood network* of each genome is built using the information about protein coding gene coordinates. Each gene is connected to the following one in the genome table, with no threshold on their distance and taking into account the circularity of the chromosome. Genes corresponding to removed proteins (see *Orthologous mapping*) are deleted at this stage by joining together predecessor and successor nodes. Taking advantage of the orthologous mapping and the gene ordering information encoded in the genome table files, all gene neighborhoods are stored in compatible adjacency matrices (the *genome specific neighborhood networks*, GSN), i.e. proteins belonging to the same OG occupy the same place in matrices corresponding to different chromosomes. The GSN is encoded as un undirected graph. Once the GSN for all genomes have been built, they can be compared on a pairwise basis (see Figure 1 and Figure 2). We call GGN the network for a given comparison, which is obtained by taking the sum of the adjacency matrices of the two GSNs under analysis. By summing all the GSNs from a group it is possible to extract connected components that are present in a given fraction of the genomes i.e. evolutionary conserved gene clusters. To this purpose we use the Dulmage-Mendelsohn decomposition performed by the matlab function dmperm.

##### Graph compression

The GSN is circular and all genes have one incoming and one outgoing edge only. The aim of the compression procedure is to remove a defined set of accessory genes (*R*) and add the connections between predecessors and successors of genes belonging to *R* (Figure 1), allowing to focus on the re-organization of the genome backbone. For each gene in *R*, we add to the graph the edge between its core predecessor and its core successor, and then we remove from the graph the genes in *R*. If part of the genes belonging to *R* form connected groups of genes, they are treated as a single gene. Genes in the compressed GSN correspond to genes common to the two genomes under comparison and consequently the compression of a given genome can be different for each comparison. To be noticed that the compressed network goes in the direction of the work of [32], and that it has a different meaning with respect to the original GSN, since after the compression, edges do not always correspond to physical proximity between genes (see Figure 2a) and cannot be used for identification of evolutionarily persistent gene clusters.

### Diameter of the graph and stability

The diameter of the networks was calculated using the MatlabBGL package developed by David Gleich, [35].

### Non linear fitting and model comparison

Several non linear functions were fitted to the data using the Curve Fitting Tool in Matlab (Mathworks Inc, r2009b) and the Trust-region algorithm. Comparisons of the estimated models were done taking advantage of the Akaike information criterion (AIC), which combines the goodness of fit of a model and a penalty on the number of parameters in a single score. It is moreover appropriate with non-nested models, which is our case. AIC for regression models is defined below:

(10) $$\text{AI}C_{i}=N\cdot \text{ln}\left(\frac{SS_{i}}{N}\right)+2\cdot K_{1},$$

where *N* is the total number of observations, *SS*_*i*_ is the total sum of squared errors for model *i*, and *K*_*i*_=1+*N*_*parameters*_. In general, the model with the lowest AIC is considered the best approximation to the data. To better quantify the plausibility of each model, it is interesting to estimate the *Akaike weights* of all models. It holds:

(11) $${ℒ}_{i}(\text{mode}l_{i}|\text{data})\propto \text{exp}(-0.5\cdot \Delta_{i}),$$

where *Δ*_*i*_ = *AIC*_*i*_ - *AIC*^*min*^. The right-hand side of the above equation is known as the *relative likelihood* of the model. The relative likelihood can be used to calculate the Akaike weights (*w*_*i*_):

(12) $$w_{i}=\frac{\text{exp}(-0.5\cdot \Delta_{i})}{\overset{R}{\underset{r=1}{\sum }}\text{exp}(-0.5\cdot \Delta_{r})},$$

where *R* is the number of models under comparison. Akaike weights inform on how much more probable is the model with the lowest AIC, with respect to the other models allowing not only to identify the best model, but also to say something on how far the others are from its performance.

### Selection of the dataset

The idea behind this analysis is twofold. On one side, we aim at studying the properties of the gene neighborhood network within each species during evolution to get information on genome stability on the short phylogenetic time. This within-species approach allows to study short-term gene order stability for each genome under analysis; for most of the species, this is a period where no major changes in life-style/ecological niche happened. We can consequently predict some homogeneity of selective pressures acting on genomes belonging to the same species. Our dataset comprises all prokaryotic species for which at least 4 genomes were available when the data were first downloaded (December 2010), for a total of 277 genomes spread over 40 species (see Table 2). This resulted in 1286 pairwise intra-species comparisons. The reason for setting this minimum number of genomes for each species is explained below.

**Table 2:** Genomic features of the species under analysis

**Organism**	**Core**	**Acc.**	**Sing.**	**Ref. (id)**	**Abbrev.**	**N**	**Pathogen**	**Taxonomy**
*Acinetobacter baumannii*	1994	1676	1889	ACICU (58765)	ABAU	6	*X*	*γ*
*Bacillus anthracis*	4318	1620	709	CDC 684 (59303)	BANT	6	*X*	Firmicutes
*Bacillus cereus*	3656	2672	3855	B4264 (58757)	BCEREU	9	*x*	Firmicutes
*Bifidobacterium longum*	1193	998	1458	NCC2705 (57939)	BLONG	7	-	Actino.
*Buchnera aphidicola*	326	252	40	Sg (57913)	BAPHI	6	-	*γ*
*Burkholderia cenocepacia*	5288	1527	2146	AU 1054 (58371)	BCENO	4	*X*	*β*
*Burkholderia mallei*	3526	1885	1963	NCTC 10229 (58383)	BMALL	4	*X*	*β*
*Burkholderia pseudomallei*	2942	3748	2871	K96243 (57733)	BPMALL	5	*X*+plant	*β*
*Campylobacter jejuni*	1005	752	911	NCTC 11168 (57587)	CJEJU	7	*X*	*ε*
*Chlamydia trachomatis*	851	47	27	Bu (61633)	CTRAC	6	*X*	Chlamydia
*Chlamydophila pneumoniae*	1020	52	147	J138 (57829)	CPNEU	4	*X*	Chlamydia
*Clostridium botulinum*	1153	3636	3201	A Hall (58931)	CBOTU	11	*X*	Firmicutes
*Coxiella burnetii*	1383	589	703	RSA 493 (57631)	CBURN	5	*X*	*γ*
*Escherichia coli*	2322	4997	7748	IAI1 (59377)	ECOLI	30	*x*	*γ*
*Francisella tularensis*	1160	512	654	OSU18 (58687)	FTULA	7	*X*	*γ*
*Haemophilus influenzae*	1130	695	542	86 028NP (58093)	HINF	6	*X*	*γ*
*Lactococcus lactis*	1566	671	1480	KF147 (42831)	LLACT	4	-	Firmicutes
*Legionella pneumophila*	2433	752	849	Paris (58211)	LPNEU	5	*X*	*γ*
*Listeria monocytogenes*	2474	623	557	EGD e (61583)	LMONO	6	*X*	Firmicutes
*Methanococcus maripaludis*	1487	225	497	C6 (58947)	MMARI	4	-	Euryarch.
*Mycobacterium tuberculosis*	3627	445	590	CDC1551 (57775)	MTUBE	5	*X*	Actino.
*Neisseria meningitidis*	1467	506	755	MC58 (57817)	NMENI	5	*X*	*β*
*Prochlorococcus marinus*	1232	1725	2027	CCMP1986 (57761)	PMARIN	12	-	Cyano.
*Pseudomonas aeruginosa*	4909	842	1694	PA7 (58627)	PAERU	4	*x*(opp.)	*γ*
*Pseudomonas putida*	3773	1279	2535	F1 (58355)	PPUT	4	-	*γ*
*Ralstonia solanacearum*	2442	2000	2698	CFBP2957 (50545)	RSOLA	4	*X* plant	*α*
*Rhodobacter sphaeroides*	2938	1177	2158	ATCC 17029 (58449)	RSPHAE	4	-	*α*
*Rhodopseudomonas palustris*	2610	2673	3516	TIE 1 (58995)	RPALU	7	-	*α*
*Salmonella enterica*	2645	2904	3506	Gallinarum 287 91 (59249)	SENT	16	*X*	*γ*
*Shewanella baltica*	3520	745	1891	OS185 (58743)	SBALT	4	-	*γ*
*Staphylococcus aureus*	1879	1166	906	Newman (58839)	SAUR	15	*X*	Firmicutes
*Streptococcus pneumoniae*	1407	1091	1137	ATCC 700669 (59287)	SPNEU	14	*X*	Firmicutes
*Streptococcus suis*	1544	478	764	P1 7 (32235)	SSUIS	6	*X*	Firmicutes
*Sulcia muelleri*	193	51	37	SMDSEM (59393)	SMUELL	4	-	Bacteroid.
*Sulfolobus islandicus*	2061	794	1152	M 16 4 (58841)	SISLA	7	-	Crenarch.
*Vibrio cholerae*	3224	595	795	M66 2 (59355)	VCHOL	4	*X*	*γ*
*Xanthomonas campestris*	3381	764	1854	ATCC 33913 (57887)	XCAMP	4	*X* plant	*γ*
*Xylella fastidiosa*	1639	542	1070	Temecula1 (57869)	XFAST	4	*X* plant	*γ*
*Yersinia pestis*	2791	1425	2158	Nepal516 (58609)	YPEST	8	*X*	*γ*
*Yersinia pseudotuberculosis*	3406	687	1339	IP 32953 (58157)	YPTUB	4	*X*	*γ*

*Core*, number of proteins common to all genomes within the species; *Acc.*, accessory proteins, present in at least 2 genomes; *Sing.*, singleton proteins, present in only one genome; *Ref.(id)*, strain used to perform inter-species comparisons and its project identifier in the NCBI Genome database; *Abbrev.* is the abbreviation used in the figures; *N* indicates the number of genomes belonging to each species; *Pathogen*, *X* are the pathogens, *x* indicate species comprising both pathogen and non pathogen strains and - is for non pathogens. Plant pathogens are also indicated and *opp.* indicates opportunist pathogens. *P. aeruginosa* was considered non-pathogen for probability calculations. *Taxonomy* is the taxonomy of the species.

On the contrary, when we consider a wider phylogenetic span for the comparisons, the probability that two genomes come from species with highly similar life-styles is reduced, and we can analyze changes in stability that occurred in ancestors of the present species (long-term stability analysis). This dataset concerns 40 genomes (one for each of the species under analysis), and comprises 780 pairwise comparisons, corresponding to 39 new observations for each reference genome.

For the purpose of statistical model fitting only, we add a set of comparisons between genomes belonging to 32 genera (see Additional file 1), and the comparisons were made only within each genus. To be noticed that this dataset is only used for statistical model fitting.

#### Minimum number of genomes *per* species

We ask for at least 4 genomes when selecting species for the short term analysis for two main reasons. First in such a way, we have some statistical power for intra-species comparisons allowing to identify deviant genomes with respect to the average species behavior. This is important if we want to identify genomes that changed their stability recently. Second, this within-species analysis allows to select the genomes for the comparisons between species. These genomes were chosen so that they have a stability which is in line with the other genomes belonging to the same species. It should be noticed that if genomes for the long term analysis are picked randomly within a species, we could end up using a biased genome as the prototypical species genome, affecting the subsequent analysis and interpretation of the results. Thus choosing genomes whose stability pattern corresponds to some sort of *average* of the species allows to obtain more accurate stability values for the species in the long-term analysis.

### Phylogenetic distances

For distance calculations, we used two universal sequences proposed by [55], namely FusA and RplB. Coding sequences were aligned at the protein level using RevTrans [56]. Distances were calculated using Mega 5 [57], Tamura-3 parameters model of evolution for DNA sequences, heterogeneous rates along lineages (*α*=1.3, default value), and the pairwise deletion option. The distance matrices obtained for the two proteins were combined together by taking the sum of the corresponding elements and used for subsequent analysis.

## Competing interests

The authors declare that they have no competing interests.

## Authors’ contributions

All authors conceived and developed the ideas behind this work. MB developed the tools for the analysis in Matlab and Java. All authors contributed to the final form of the paper. All authors read and approved the final manuscript.

## Supplementary Material

Additional file 1 **The Genus dataset.**The genus dataset allowed to increase the number of comparisons for parameter identification. Click here for file

Additional file 2 **Cyanobacteria.** Comparisons within the *P. marinus* species and of members of two other cyanobacterial genera: *Synechococcus* (×) and *Cyanothece* (∘). The comparisons between *P. marinus* strains give on average larger stability values than for the other comparisons that cannot be explained by the different phylogenetic distances in the comparisons. If all these genomes were compared as a group, it would be more difficult, if not impossible, to discern the higher stability of *P. marinus*. Click here for file

Additional file 3 **Relationship between backbone and genome organization stability by species.** The species specific relationship between GOS and BS. In the title we report the abbreviated name of the species and the regression coefficient. The size of the markers is proportional to the average phylogenetic distance within the species. Click here for file

Additional file 4 **Relationship between genome organization stability and genomic fluidity by species.** Relationship between genome organization stability (GOS) and genomic stability (*σ*). Plots are in double logarithmic scale. Click here for file

Additional file 5 **Distribution of singleton size by species.** Distribution of the size of singleton components. Plots are in double logarithmic scale; x-axis is the length of the singleton gene clusters, y-axis is the absolute abundance. Click here for file

Additional file 6 **Removed proteins are mostly mobile elements.** Most of the proteins removed in the pre-processing step have significant similarity to proteins in the Aclame database containing mobile elements [58,59]. Click here for file
