# Supplementary material for: Short and long-term genome stability analysis of prokaryotic genomes
Source: BMC Genomics. 2013 May 8;14:309. doi: 10.1186/1471-2164-14-309 (PMC3683328; doi:10.1186/1471-2164-14-309)
Supplement: Additional file 1 — The Genus dataset.The genus dataset allowed to increase the number of comparisons for parameter identification. [file 1471-2164-14-309-S1.pdf]

**Genus**	**N genomes**
Bartonella	6
Bordetella	5
Borrelia	8
Bradyrhizobium	3
Brucella	10
Caldicellulosiruptor	7
Campylobacter	11
Chlorobium	7
Corynebacterium	10
Cyanothece	6
Dehalococcoides	5
Desulfovibrio	8
Dickeya	4
Ehrlichia	5
Geobacillus	7
Geobacter	8
Leptospira	6
Methanocaldoccus	5
Methanococcus	7
Methylobacterium	8
Mycoplasma	27
Neisseria	8
Paenibacibacillus	4
Pyrobaculum	4
Pyrococcus	4
Rhizobium	6
Rhodococcus	4
Rickettsia	13
Synechococcus	11
Thermococcus	5
Thermotoga	15
Thermus	3
