# Supplementary figures and images for: Short and long-term genome stability analysis of prokaryotic genomes
Source: BMC Genomics. 2013 May 8;14:309. doi: 10.1186/1471-2164-14-309 (PMC3683328; doi:10.1186/1471-2164-14-309)

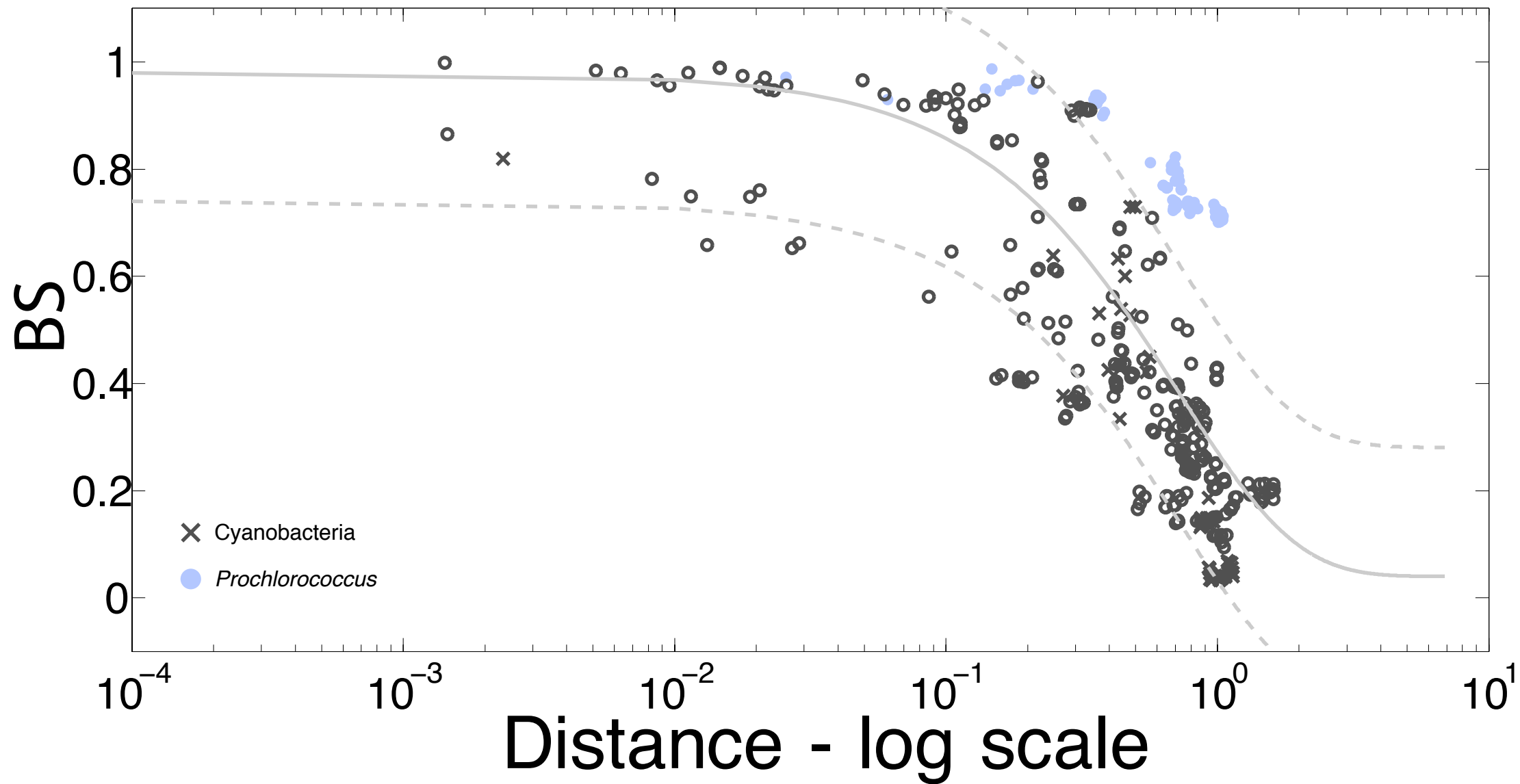

Supplement: Additional file 2 — Cyanobacteria. Comparisons within the P. marinus species and of members of two other cyanobacterial genera: Synechococcus (×) and Cyanothece (∘). The comparisons between P. marinus strains give on average larger stability values than for the other comparisons that cannot be explained by the different phylogenetic distances in the comparisons. If all these genomes were compared as a group, it would be more difficult, if not impossible, to discern the higher stability of P. marinus. [file 1471-2164-14-309-S2.pdf]

BS

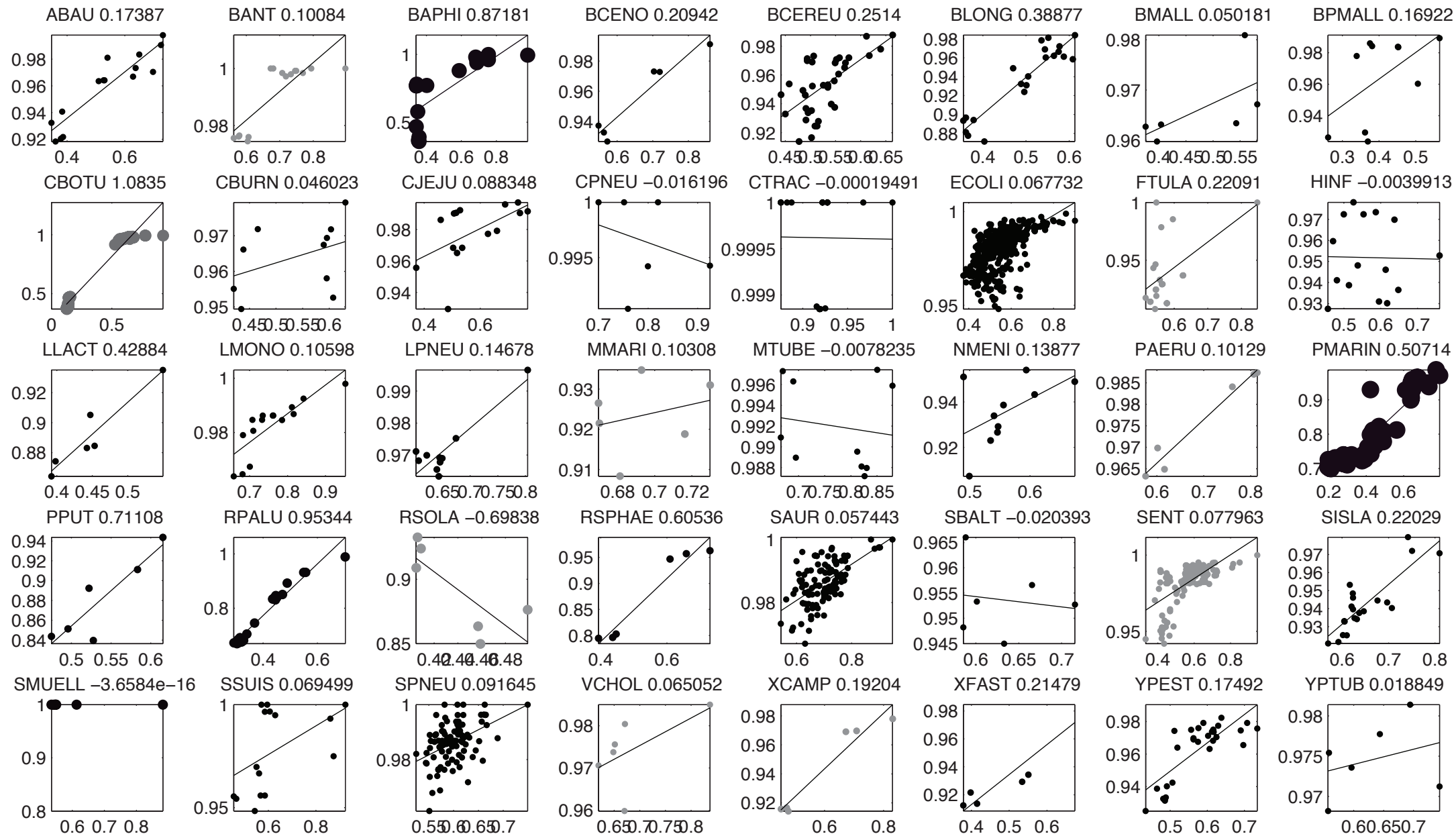

GOS

Supplement: Additional file 3 — Relationship between backbone and genome organization stability by species. The species specific relationship between GOS and BS. In the title we report the abbreviated name of the species and the regression coefficient. The size of the markers is proportional to the average phylogenetic distance within the species. [file 1471-2164-14-309-S3.pdf]

$\log_{10}(\text{GOS})$

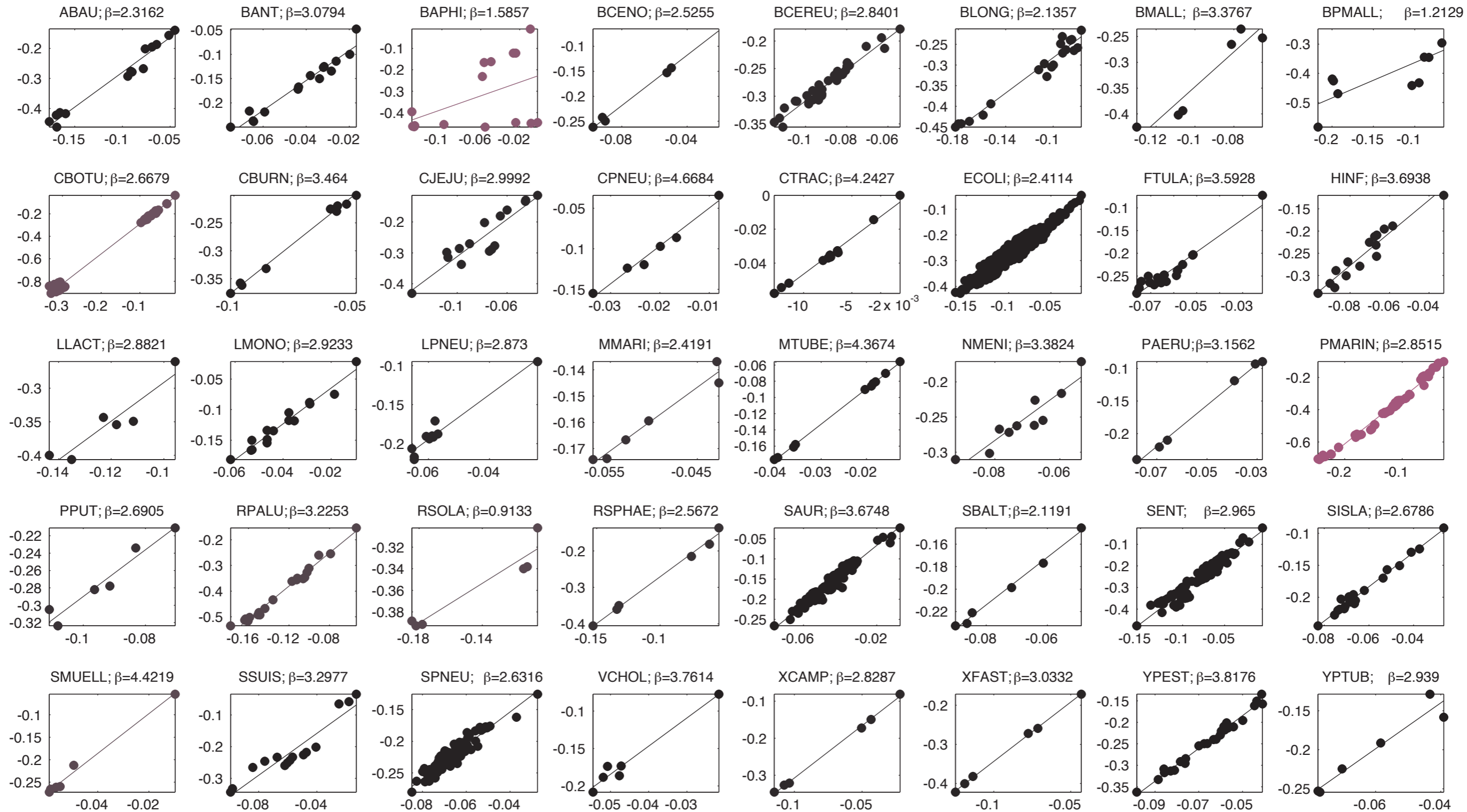

$\log_{10}(\sigma)$

Supplement: Additional file 4 — Relationship between genome organization stability and genomic fluidity by species. Relationship between genome organization stability (GOS) and genomic stability (σ). Plots are in double logarithmic scale. [file 1471-2164-14-309-S4.pdf]

N

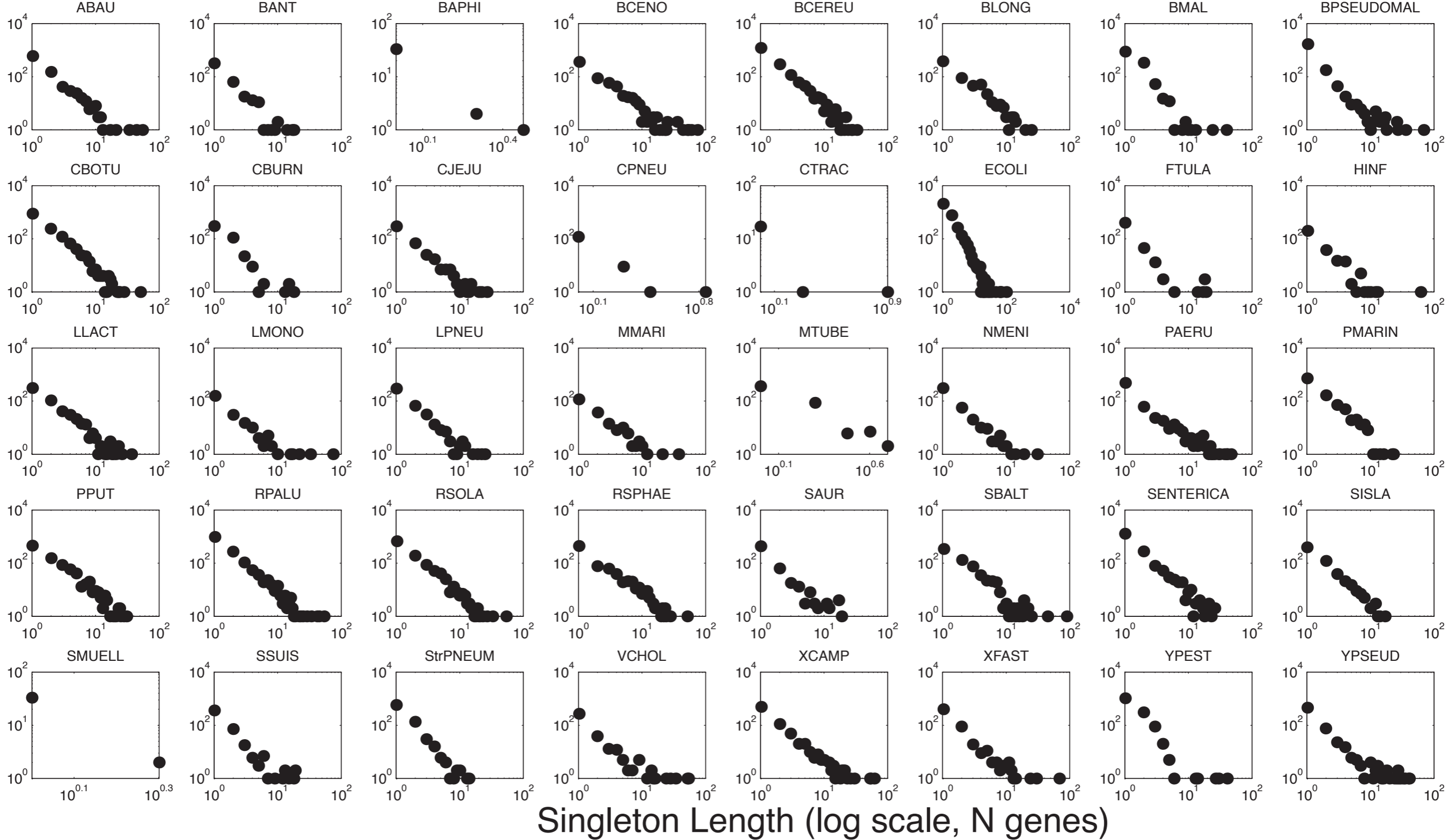

Supplement: Additional file 5 — Distribution of singleton size by species. Distribution of the size of singleton components. Plots are in double logarithmic scale; x-axis is the length of the singleton gene clusters, y-axis is the absolute abundance. [file 1471-2164-14-309-S5.pdf]
