# Supplementary material for: Short and long-term genome stability analysis of prokaryotic genomes
Source: BMC Genomics. 2013 May 8;14:309. doi: 10.1186/1471-2164-14-309 (PMC3683328; doi:10.1186/1471-2164-14-309)
Supplement: Additional file 6 — Removed proteins are mostly mobile elements. Most of the proteins removed in the pre-processing step have significant similarity to proteins in the Aclame database containing mobile elements [58,59]. [file 1471-2164-14-309-S6.pdf]

**Species**	**Fraction mobile**	**Average removed**
Acinetobacter baumannii	0.87	99
Bacillus anthracis	0.74	34
Bacillus cereus	0.79	224
Bifidobacterium longum	0.82	80
Buchnera aphidicola	1	2
Burkholderia cenocepacia	0.94	55
Burkholderia mallei	0.85	47
Burkholderia pseudomallei	0.91	69
Campylobacter jejuni	0.62	37
Chlamydia trachomatis	NA	0
Chlamydophila pneumoniae	NA	0
Clostridium botulinum	0.86	397
Coxiella burnetii	0.48	13
Escherichia coli	0.87	402
Francisella tularensis	0.71	20
Haemophilus influenzae	0.9	63
Lactococcus lactis	0.87	76
Legionella pneumophila	0.80	44
Listeria monocytogenes	0.68	17
Methanococcus maripaludis	0.43	16
Mycobacterium tuberculosis	0.47	3
Neisseria meningitidis	0.55	28
Prochlorococcus marinus	0.50	53
Pseudomonas aeruginosa	0.77	26
Pseudomonas putida	0.84	73
Ralstonia solanacearum	0.96	121
Rhodobacter sphaeroides	0.99	77
Rhodopseudomonas palustris	0.90	304
Salmonella enterica	0.91	286
Shewanella baltica	0.99	52
Staphylococcus aureus	0.72	105
Streptococcus pneumoniae	0.59	108
Streptococcus suis	0.67	20
Sulcia muelleri	0.75	1
Sulfolobus islandicus	0.69	94
Vibrio cholerae	0.61	5
Xanthomonas campestris	0.62	31
Xylella fastidiosa	0.85	51
Yersinia pestis	0.91	71
Yersinia pseudotuberculosis	0.83	41
